# Distinct inter-domain interactions of dimeric versus monomeric α-catenin link cell junctions to filaments

**DOI:** 10.1038/s42003-023-04610-x

**Published:** 2023-03-16

**Authors:** Erumbi S. Rangarajan, Emmanuel W. Smith, Tina Izard

**Affiliations:** 1 The Cell Adhesion Laboratory, UF Scripps, Jupiter, FL 33458 USA; 2 The Skaggs Graduate School, The Scripps Research Institute, Jupiter, FL 33458 USA

**Keywords:** Cryoelectron microscopy, Adherens junctions

## Abstract

Attachment between cells is crucial for almost all aspects of the life of cells. These inter-cell adhesions are mediated by the binding of transmembrane cadherin receptors of one cell to cadherins of a neighboring cell. Inside the cell, cadherin binds β-catenin, which interacts with α-catenin. The transitioning of cells between migration and adhesion is modulated by α-catenin, which links cell junctions and the plasma membrane to the actin cytoskeleton. At cell junctions, a single β-catenin/α-catenin heterodimer slips along filamentous actin in the direction of cytoskeletal tension which unfolds clustered heterodimers to form catch bonds with F-actin. Outside cell junctions, α-catenin dimerizes and links the plasma membrane to F-actin. Under cytoskeletal tension, α-catenin unfolds and forms an asymmetric catch bond with F-actin. To understand the mechanism of this important α-catenin function, we determined the 2.7 Å cryogenic electron microscopy (cryoEM) structures of filamentous actin alone and bound to human dimeric α-catenin. Our structures provide mechanistic insights into the role of the α-catenin interdomain interactions in directing α-catenin function and suggest a bivalent mechanism. Further, our cryoEM structure of human monomeric α-catenin provides mechanistic insights into α-catenin autoinhibition. Collectively, our structures capture the initial α-catenin interaction with F-actin before the sensing of force, which is a crucial event in cell adhesion and human disease.

## Abstract (web summary)

CryoEM structures capture the initial α-catenin interaction with F-actin before the sensing of force.

## Introduction

Multicellular organisms have well-defined and tightly regulated mechanisms for cell adhesion. Specifically, their endothelial, epithelial, and neuronal tissues require specialized cell-cell adhesion complexes, termed adherens junctions. These junctions are crucial for several normal functions, including embryonic morphogenesis, tissue integrity, homeostasis, and wound healing^1,2^. Disruption of adherens junctions often leads to the development of cancer and vascular diseases^3,4^. Specifically, the disassembly of adherens junctions causes loss of cell polarity and contact inhibition that favor the epithelial-to-mesenchymal transition^5–7^. Such disruption of the epithelial polarity or cell-cell contacts often results in developmental defects^8–12^. Nevertheless, for cells to move and migrate, these specialized cell-cell junctions require dynamic regulation to allow continuous formation and severance of adhesive interactions between neighboring cells^2,13^.

The cadherin-associated macromolecular complex is the master cell adhesion regulator that links neighboring cells^14,15^. Cadherin is a transmembrane protein that dimerizes with cadherin of an adjacent cell and also binds β-catenin intracellularly. β-Catenin then binds the α-catenin to form the cadherin/β-catenin/α-catenin heterotrimer^1,16^. This complex mechanically couples neighboring cells to their respective underlying actin cytoskeleton^14,17^. The actin cytoskeleton is a key regulator, and its alterations control the morphogenetic movements of sheets of cells. Through its interactions with α-catenin, the cytoskeleton controls many cellular functions such as cell motility, cell shape, cell polarity, or the regulation of transcription.

In addition to providing cell-cell adhesion, binding of the cadherin/β-catenin/α-catenin heterotrimer to the actin cytoskeleton strengthens tissues mechanically^18,19^. This protein complex also senses and transmits mechanical tension between cells during collective cell migration^13^. Such mechanical forces are either external, for example, the shearing forces of flowing blood in vessels, or inside the cells whereby myosin motor proteins induce contraction of the actin cytoskeleton^13,20–22^. α-Catenin regulates the polymerization of the cytoskeleton that transitions cells between migration and adhesion. Thus, not surprisingly, α-catenin knockout mice display defective cell adhesion, migration, and proliferation^23^.

α-Catenin is highly dynamic and functions either as a monomer or dimer. Both quaternary α-catenin states bind the actin cytoskeleton, although with different affinities that are increased by cytoskeletal tension^24–27^. However, only monomeric α-catenin binds β-catenin since the homo- and heterodimer binding sites overlap on the α-catenin amino-terminal domain. As a dimer, α-catenin resides in the cytosol and strongly induces filopodia, vital structures that initiate epithelial cell-cell contacts^28,29^ in contrast to undecorated F-actin, which is mostly limited to lamellipodia. In addition to regulating the organization of F-actin^30^, the α-catenin dimer also connects the plasma membrane to the actin cytoskeleton, thereby promoting cell adhesion and cell migration^28^. Mammalian α-catenin binds and bundles actin filaments^30^ and inhibits the seven-subunit actin-related protein2/actin-related protein 3 (Arp2/3)-mediated nucleation of F-actin assembly^26^. Depleting cytosolic α-catenin dimers increases the actin-dependent membrane dynamics and the cell-migration rate of Madin-Darby Canine Kidney epithelial cells^31^.

Binding studies have relied on α-catenin pull-downs with F-actin, which are difficult to interpret because of the tendency of α-catenin to precipitate and because α-catenin transitions between a monomer and a dimer. Structures of α-catenin have mainly used isolated α-catenin domains because of the flexibility of α-catenin and the monomer-dimer transition of α-catenin that hinder high-resolution structure determinations. We initially reported the human dimeric α-catenin Δ1-81 crystal structure (PDB entry 4igg) to 3.7 Å resolution^32^. Surprisingly, the carboxy-terminal α-catenin F-actin binding domains were not related by the two-fold axis that relates the remainder of the two α-catenin polypeptide chains. An apparently monomeric full-length murine α-catenin dimerized in the crystal and showed disorder for the amino-terminal and the carboxy-terminal domain and provided 6.5 Å insights for residues 85–262 and 290–631 (out of 906 residues) (PDB entry 4k1n)^33^. Another 4 Å murine dimeric truncated α-catenin (Δ1–81 and Δ884–906) crystal structure lacked interpretable electron density for the F-actin binding domain (PDB entry 6o3e)^34^. Therefore, any structural data on monomeric α-catenin is long overdue to fill this large knowledge gap. Here we report the 6.9 Å monomeric α-catenin cryogenic electron microscopy (cryoEM) structure. To the best of our knowledge, this is the first report of full-length α-catenin in its monomeric state that provides mechanistic insights into monomeric α-catenin function. Our data suggest that the linker preceding the α-catenin carboxy-terminal F-actin binding domain (FABD) forms an α-helix that restricts the flexibility of the FABD in monomeric α-catenin. Unexpectedly, monomeric α-catenin shows amino-terminal (instead of the previously suggested carboxy-terminal) flexibility, which has different functional consequences, and therefore provides structural clues about its autoinhibition in particular with respect to binding to F-actin.

Further, α-catenin functions outside cell junctions where dimeric α-catenin modulates F-actin organization^30^ and regulates cell migration and cell adhesion by simultaneously binding to F-actin and the plasma membrane^28^. Recently, the cryoEM structures of the isolated α-catenin FABD bound to F-actin provided insights into monomeric α-catenin^35,36^, as envisioned in a heterodimeric complex with β-catenin, at cell junctions. Here we report the much higher resolution (2.8 Å) cryoEM structure of human dimeric α-catenin bound to F-actin and the 2.7 Å cryoEM structure of F-actin. Surprisingly, the α-catenin FABDs are detached from the remainder of the molecule. Functionally important, the lack of interdomain interactions suggests that the amino-terminal α-catenin domains of dimeric α-catenin might bind to the plasma membrane independently of the free α-catenin FABD engaging in interactions with filamentous actin. Our data also suggest a bivalent mechanism. Collectively, our results advance our knowledge of how α-catenin functions allosterically to regulate specific signaling pathways depending on its oligomeric state.

## Results

### Human monomeric α-catenin has distinct interdomain interactions

**Fig. 1:**
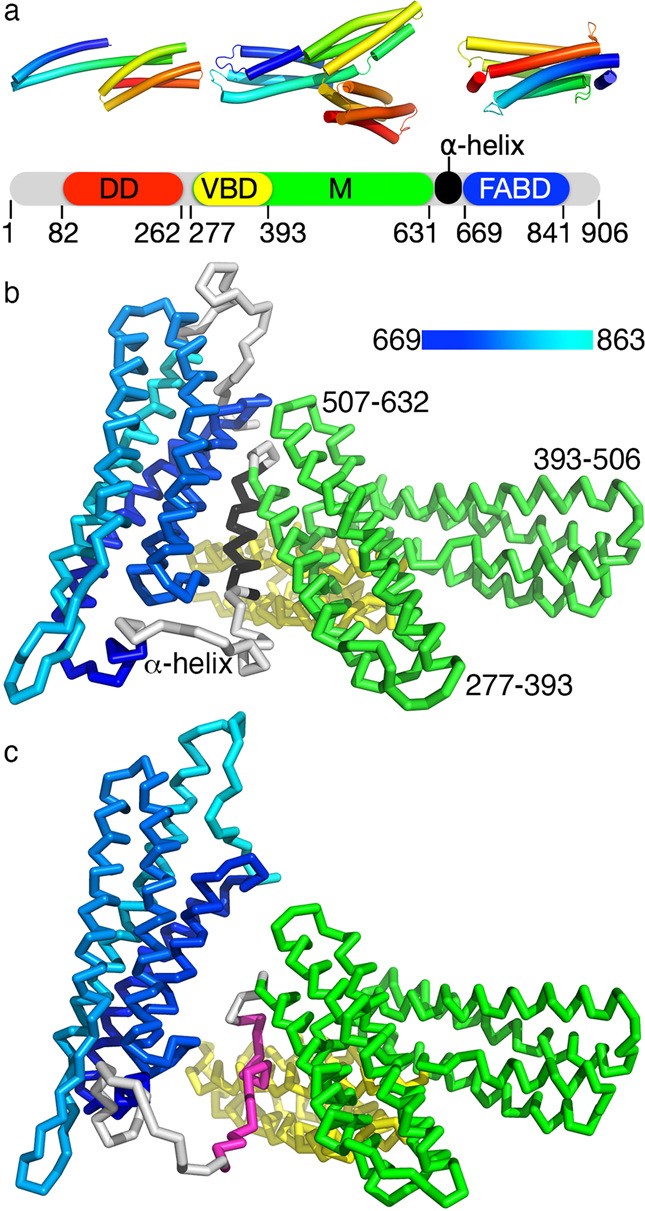
The monomeric α-catenin cryoEM structure reveals interdomain interactions with the F-actin binding domain (FABD) and an ordered α-helix. **a** Domain structure of α-catenin. Top structures of the individual domains are colored spectrally from shorter to longer wavelengths. Bottom, domain boundaries are shown and indicated by their residue numbers (DD, dimerization domain; VBD, vinculin binding domain; M, middle domain; FABD, F-actin binding domain). The VBD is part of the middle domain. The FABD is a 5-helix bundle plus two short α-helices on either end. **b** Cartoon of our monomeric α-catenin cryoEM structure. The VBD is shown in yellow, the remainder of the middle domain is in green, and the carboxy-terminal FABD is colored blue to cyan for residues 669 to 863. The linker residues (636-650, black) that form an unexpected α-helix are indicated. **c** The starting AlphaFold model does not have the extra α-helix (magenta) and the FABD (colored from blue to cyan) is further away from the middle domain (green) compared to our final refined model.

**Fig. 2:**
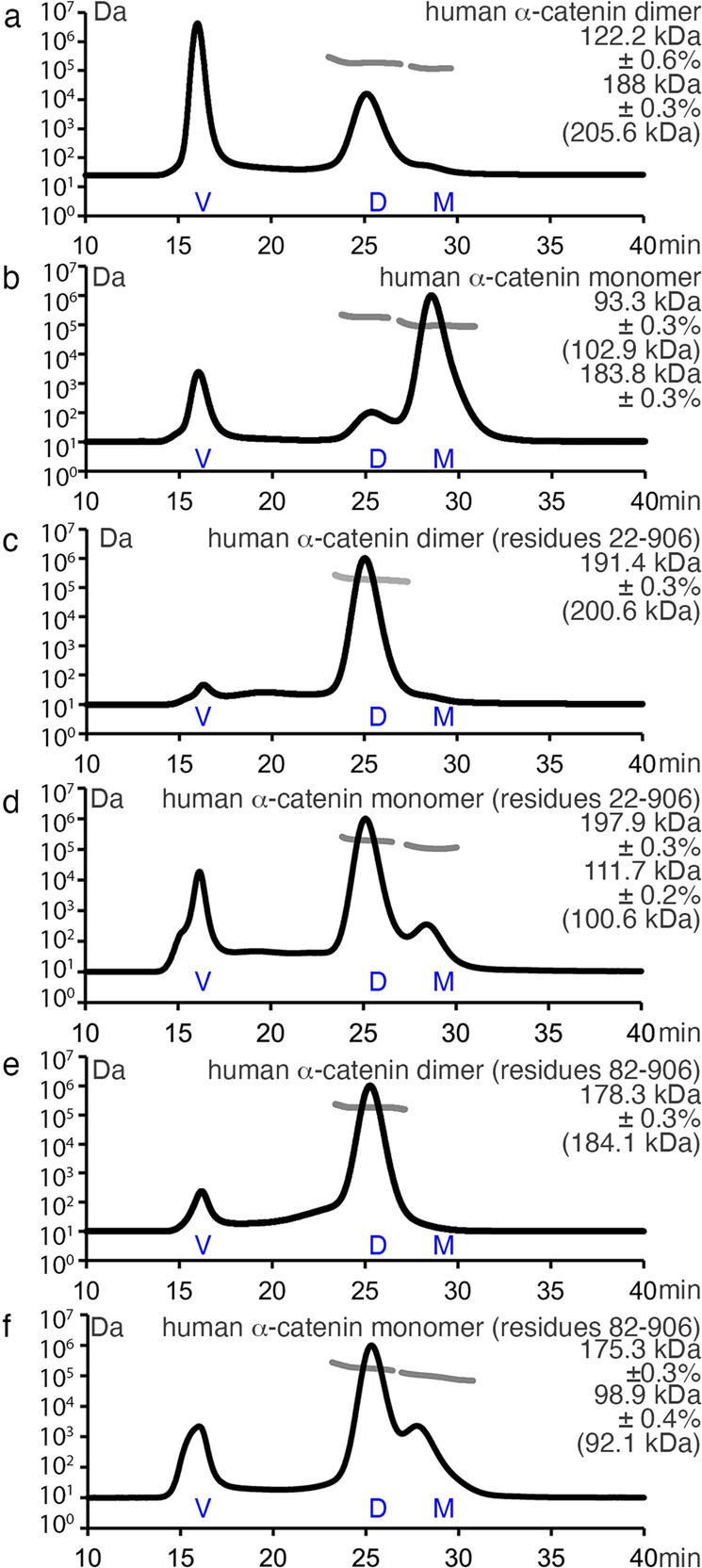
Determination of absolute masses by size exclusion chromatography and multi-angle light scattering. Full-length human α-catenin forms a dimer and monomer. Separate pooling of monomeric or dimeric peaks from a size exclusion chromatography run was subsequently analyzed by size exclusion chromatography multi-angle light scattering. The ordinate indicates the molar mass (light gray trace) expressed on a logarithmic scale, and the abscissa represents the time in minutes for the light scattering profile (black trace). The ordinate corresponding to the light scattering signal is not shown. The experimental absolute masses are shown. Values in brackets are the molecular weights as calculated from the respective polypeptide chains. **a** Human dimeric α-catenin, **b** human monomeric α-catenin, **c** human dimeric α-catenin Δ1–21, **d** human monomeric α-catenin Δ1–21, **e** human dimeric α-catenin Δ1–81, and **f** human monomeric α-catenin Δ1–81.

To gain molecular and mechanistic insights into the function of monomeric α-catenin, we solved the cryoEM structure of monomeric α-catenin to 6.9 Å resolution (Fig. 1 and Supplementary Fig. S1). Our sample comprises full-length monomeric α-catenin (residues 1–906) as we confirmed by size exclusion chromatography multi-angle light scattering (Fig. 2 and Supplementary Table S1).

Surprisingly, in our structure, the two amino-terminal 4-helix bundle domains (residues 1–276) are disordered and not visible (Fig. 1b) which explains the modest resolution despite countless efforts that, for example, included treatment with glutaraldehyde. Nevertheless, we posit that this structure determination is a substantial achievement given that attempts to determine the monomeric α-catenin structure proved too difficult for the past decade^33^. Given the wealth of available crystal and AlphaFold^37^ structures, insights are obtained even at this modest resolution. For example, our monomeric α-catenin uncovered interactions of the middle domain with the FABD that have not been seen in any of the reported crystal structures. Specifically, the FABD interacts with the last 4-helix bundle of the middle domain through hydrophobic interactions. Charged residues surround the domain interface that might contribute towards the conformational flexibility of the full-length protein. In addition, the α-helix that we observe in the linker region that connects the middle domain to FABD (residues 636-650) is positioned centrally. This central location places this α-helix near the vinculin binding domain, the last 4-helix bundle of the middle domain, and the FABD. Therefore, this α-helix might play a role in the interdomain interactions that are severed depending on the functional state of α-catenin. Finally, the carboxy-terminal 43 residues were found to be disordered and could not be modeled.

The observed amino-terminal disorder is consistent with size-exclusion chromatography small-angle X-ray scattering (SEC-SAXS) data that showed that the α-catenin amino-terminal domain connects *via* a flexible linker (residues 262–277) that undergoes motions relative to the α-catenin middle domain^38^. In our previously reported dimeric crystal structure, the carboxy-terminal FABDs were oriented differently to engage in distinct inter-domain interactions with the first 4-helix bundle domain (residues 277–392) of the middle domain that binds to vinculin^32^ (Supplementary Fig. S2). These interdomain contacts are not seen in our monomeric α-catenin cryoEM structure. Instead, the FABD of monomeric α-catenin engages in interactions only with the last 4-helix bundle of the middle domain (residues 507–632) (Fig. 1c, d; Supplementary Table S2). Notably, residues 636-650 that connect the middle domain to the FABD form an α-helix that has not been ordered in any α-catenin structure thus far. This 636–650 α-helix lies almost parallel to the last 4-helix bundle of the middle domain (residues 507–631) (Fig. 1c). The 636-650 connecting α-helix is also near the vinculin binding domain (VBD) where, for example, residue D649 is in proximity with VBD residue R332 (Supplementary Fig. S2d).

### Dimeric α-catenin binding to filamentous actin

**Fig. 3:**
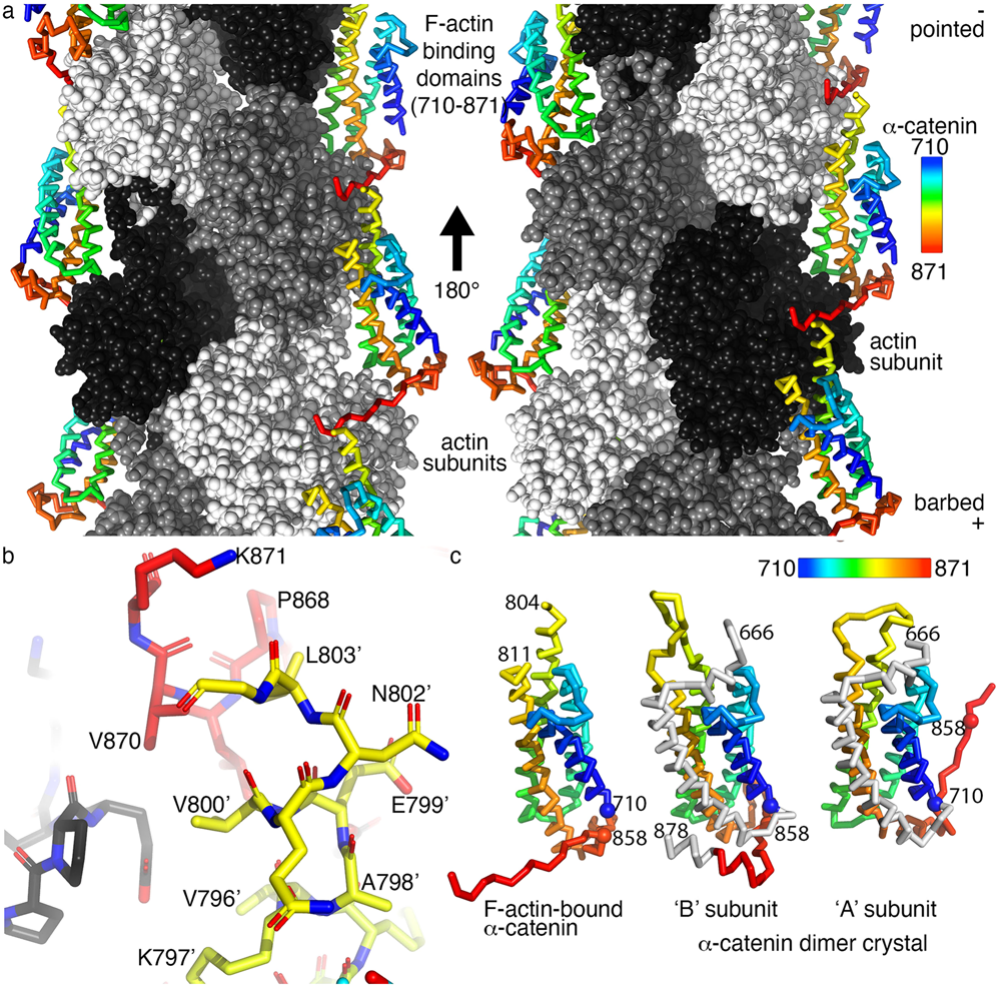
The 2.8 Å cryoEM structure of dimeric α-catenin bound to F-actin. **a** Cα trace of α-catenin, colored spectrally from shorter to longer wavelengths for residues 710–871 as indicated. Actin subunits are shown as white, gray, or black spheres. The left and right panels are views rotated 180° relative to each other as indicated. The F-actin barbed and pointed ends are indicated. **b** Close-up view of the α-catenin/α-catenin interaction, colored spectrally. The distance from V870 to V800’ and L803’ is about 4 and 4.9 Å, respectively, while L869 with V796’ is about 3.9 Å. The prime indicates a residue on a separate α-catenin polypeptide chain. **c** Cα trace of the α-catenin FABD bound to F-actin (left) compared to the FABD as seen in the two protomers in the unbound α-catenin dimer crystal structure (middle and right, respectively), shown in the same orientation. Terminal residues are labeled. Spheres are shown for the Cα of residues 710 and 858 to highlight their different environments.

To gain molecular and mechanistic insights into the important functions of dimeric α-catenin outside the cell junction area, we determined the 2.8 Å cryoEM structure of the human Δ1–21 α-catenin dimer bound to F-actin (Fig. 3; Supplementary Figs. S3, S4; Table 1). Disappointing at first glance, the first 709 and last 35 α-catenin residues (and a loop, α-catenin residues 805-810) are disordered in our α-catenin bound to F-actin structure. The amino-terminal disorder is consistent with size exclusion chromatography small-angle X-ray scattering data that showed that in the α-catenin dimer, the FABDs are separated from the rest of the molecule^38,39^. However, the finding that the amino-terminal domains are disordered and therefore not in contact with the α-catenin FABDs is functionally important, as it makes the FABDs readily available for binding to F-actin and thus explains its higher affinity. Our dimeric α-catenin cryoEM structure bound to F-actin and dimeric α-catenin crystal structure are also consistent with small-angle neutron scattering data that showed that the dimeric α-catenin is more extended compared to unbound dimeric α-catenin^38^.

**Table 1:** CryoEM data and protein structure refinement statistics.

	F-actin	F-actin/α-catenin	α-catenin
data collection			
magnification	60,000	60,000	60,000
total dose (e *per* Å^2^)	60	60	60
fractions	50	50	50
pixel size (Å *per* pixel)	0.72	0.72	0.72
defocus range	−0.6 to −2.6 μm	−0.6 to −2.6 μm	−0.6 to −2.6 μm
data processing			
number of micrographs	7427	7427	13,634
number of particles	963,102	403,663	154,710
helical rise	27.4 Å	27.5 Å	
helical twist	−166.7°	−166.7°	
resolution	2.7 Å	2.8 Å	6.91 Å
model refinement			
polypeptide chains	6	12	
number of non-H atoms	17,556	24,591	
protein residues	2,220	3,156	
water molecules	60	21	
magnesium ions	6	6	
bonds (r.m.s.d.)			
length (# over 4σ)	0.008 Å (12)	0.011 Å (0)	
angles (# over 4σ)	1.368° (9)	1.225° (0)	
MolProbity score	1.17	1.18	
clash score	2.48	2.62	
Ramachandran plot			
outliers	0	0	
allowed	0.0274	0.0271	
favored	0.9726	0.9729	
rotamer outliers	0	0	
Cβ outliers	0.0029	0	
CaBLAM outliers	0.0055	0.0078	
EMDB identifier	EMD-26860	EMD-26772	EMD-27717
PDB identifier	7uxf	7utj	

Refinement statistics for unbound F-actin and F-actin bound by dimeric α-catenin. All data sets were collected on a JEOL cryoARM300, at 300 kV, on a GATAN K3 detector.

Most α-catenin contacts with F-actin are formed by the second last two α-helices of α-catenin (residues 772–841) with two actin subunits. The regions corresponding to the fourth (residues 785-804) and fifth (residues 811–824) α-helices of the α-catenin FABD interact exclusively with the third subdomain (residues 145–180 and 270–337) of one actin subunit. The remaining α-catenin FABD interactions with F-actin occur predominantly with the longitudinal neighboring actin subunit. Such intermolecular interactions include a portion of fourth α-catenin FABD α-helix (residues 772–786) and regions of fifth α-helix (residues 834–841) with the actin DNAse I binding loop (D-loop). The most carboxy-terminal α-catenin region (residues 862–870) extends to bind the longitudinal neighboring actin subunit (Supplementary Table S3). Interestingly, the α-catenin carboxy terminus engages in intermolecular interactions with the fourth α-helix of the FABD helical bundle of a neighboring α-catenin molecule (Fig. 3b). This α-catenin/α-catenin interaction is formed by hydrophobic intermolecular contacts that include V870 with V800’ and L803’ or L869 with V796’ (where the prime indicates a residue on a separate α-catenin polypeptide chain) (Fig. 3b).

As noted for the murine structures^35,36^ of the isolated α-catenin FABDs bound to F-actin, we find a release of the amino-terminal FABD α-helix upon binding to F-actin. We find that this α-helix is fully exposed. In contrast, the earlier reports suggested that almost two turns of this α-helix remain in its unbound conformation.

### Structural alterations in dimeric α-catenin upon binding to F-actin

The murine dimeric α-catenin structures have their FABDs disordered and have already been compared to our human dimeric α-catenin structure that has its FABDs ordered and thus visible^35,36^. Comparison of our unbound dimeric α-catenin structure (PDB entry 4igg)^32^ with our human α-catenin bound to F-actin (this report) instead, therefore, provides new insights. In contrast to our F-actin-bound cryoEM structure (Fig. 3b), the carboxy-termini of each protomer in our dimeric unbound α-catenin crystal structure do not interact with each other^32^. In our dimeric unbound α-catenin crystal structure, each carboxy terminus has a unique conformation (Fig. 3c). In our actin-bound cryoEM α-catenin structure and in subunit ‘A’ of our unbound α-catenin crystal structure, the carboxy terminus is extended, although in different directions (Fig. 3c; Supplementary Fig. S5a–c). For example, in subunit ‘A’, α-catenin residue N710 resides near S851 (Supplementary Fig. S5a) while in subunit ‘B’ and our actin-bound structure, N710 interacts further down the polypeptide chain and resides near S858 instead (Supplementary Fig. S5b, c). However, binding to filamentous actin results in the α-catenin carboxy terminus (residues 860–862) occupying the space filled by the first α-helix of the FABD in the unbound α-catenin crystal structure that becomes disordered in the actin-bound structure (Supplementary Fig. S5d).

Differences in the two protomers in the crystal structure are also seen in the loop (residues 805–810) that connects the last two α-helices of the FABD. These are disordered in our F-actin-bound α-catenin cryoEM structure. Here, induced-fit causes α-catenin movements of ~19 Å of the carboxy-terminal half of the penultimate α-helix of the FABD 5-helix bundle (Supplementary Fig. S5e).

### Comparison of monomeric and dimeric α-catenin bound to F-actin

**Fig. 4:**
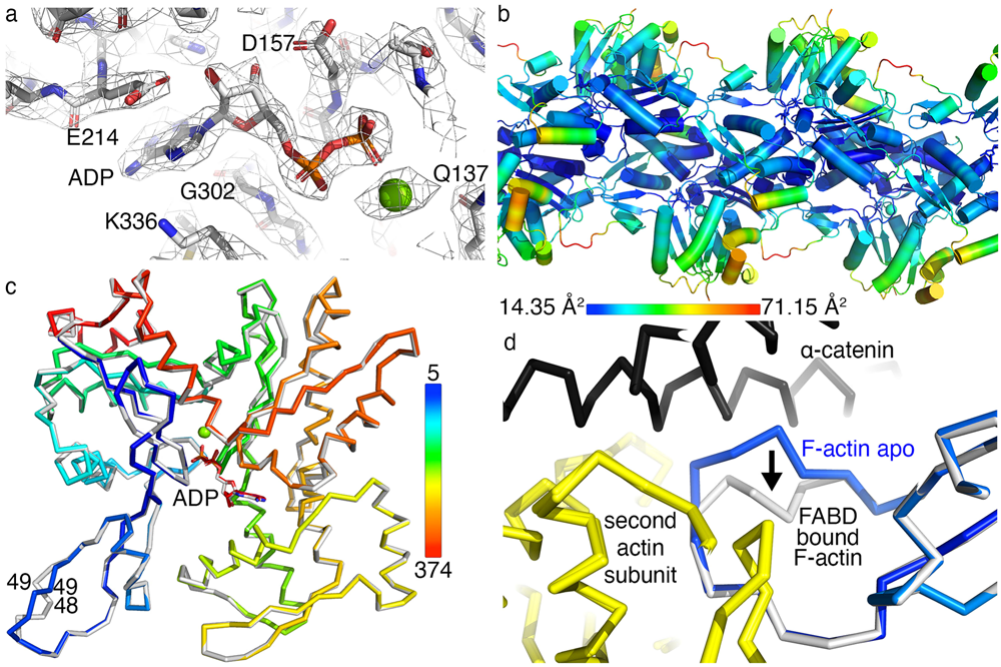
The 2.7 Å cryoEM structure of F-actin. **a** Coulomb potential map showing adenosine diphosphate binding. Green sphere, magnesium. **b** The cryoEM structure of F-actin shows the temperature factors as indicated. **c** Superposition of unbound F-actin (colored spectrally as indicated) onto F-actin when bound to α-catenin (gray). Differences in the D-loop are indicated by the numbering of some residues in that region (residues 48 and 49). The two small actin subdomains^66^ are comprised of (i) residues 1–32, 70–144, and 338–372 and (ii) (residues 33–69) while the two large subdomains harbor (iii) residues 145–180 and 270–337, and (iv) residues 181–269. **d** Close-up view of the superimposed unbound (yellow) and α-catenin bound (white) F-actin structure. α-Catenin is shown in black in the complex structure. Upon binding to α-catenin, the Cα of G48 of F-actin moves by about 7 Å (indicated by the arrow).

The isolated α-catenin FABD bound to F-actin structure seems to be a good indicator of how the α-catenin FABD interacts with F-actin when α-catenin is bound to β-catenin. These structures of the isolated (murine or human) α-catenin FABDs (residues 699–871) bound to (chicken or rabbit) F-actin^35,36,40^ (PDB entries 6wvt or 6upv, respectively) are similar to our dimeric α-catenin bound to F-actin structure and can be superimposed with root mean square deviations of 0.7 Å for 9745 atoms.  At our 2.8 Å resolution, we can fit the amino acids clearly into our obtained Coulomb potential map (Fig. 4a), which shows the electrostatic interaction of α-catenin K797 with actin E334. We also find an interaction between α-catenin Q789 and actin G146. Thus, our high-resolution structures of dimeric α-catenin (residues 22–906) bound to F-actin and F-actin alone provide detailed structural information that advance our understanding of the initial interactions of α-catenin with F-actin before the application of force.

### Structural alterations in F-actin upon binding to dimeric α-catenin

To determine the effects of α-catenin binding on the structure of F-actin, we determined the 2.7 Å cryoEM structure of F-actin (Fig. 4, Supplementary Fig. S3, and Supplementary Table S3) from the same grid and data set that resulted in our F-actin cryoEM structure bound to α-catenin. Therefore, this comparison is directly indicative of any alterations in F-actin resulting from α-catenin binding *versus* using other F-actin structures derived from a separate actin batch that could be used for such a comparison. Superposition of the actin subunits of α-catenin bound and unbound actin displays a root mean square deviation of 0.7 Å for 2888 atoms. The smallest and most flexible of the four actin domains harbors the so-called D-loop, for DNase I binding loop (residues 40–51). This loop changes its conformation upon adenosine triphosphate hydrolysis and mediates longitudinal actin-actin interactions^41,42^. Binding of α-catenin places its penultimate FABD α-helix against the D-loop, causing a shift of about 7 Å of the D-loop (Fig. 4c, d). Such D-loop conformation has also been observed in the metavinculin-bound structure (PDB entry 6upw).

## Discussion

The balance between cell migration and cell-cell adhesion is crucial for cells to migrate to specific sites during development. Upon contact with each other, cells become stationary and differentiate into tissues for which strong cell-cell adhesion is necessary to maintain tissue integrity. Cell migration requires changes in cell-cell adhesion to allow cell turnover, which, for example, is essential for wound healing. The interaction between α-catenin and the actin cytoskeleton is key to these cell adhesion processes.

α-Catenin functions allosterically to regulate specific signaling pathways depending on its oligomeric state. By binding to the cadherin/β-catenin heterodimer, monomeric α-catenin is part of the mechanosensing complex involved in force transmission. In contrast, dimeric α-catenin is recruited to the plasma membrane, where its simultaneous binding to F-actin promotes cell adhesion and migration^28^. While the α-catenin dimer binds filamentous actin readily, the cadherin/β-catenin/α-catenin heterotrimer binds F-actin with significantly lower affinity in vitro^18,25,43^. However, under tension, the cadherin/β-catenin/α-catenin heterotrimer binds F-actin strongly by a catch bond mechanism^44,45^. New data^27^ suggested that dimeric α-catenin engages in a directionally asymmetric catch bond with F-actin while the interaction of α-catenin bound to β-catenin occurs by a slip bond. While single-particle analyses cannot capture the interactions under force, our cryoEM structures identify the dimeric α-catenin interactions that occur with F-actin before the sensing of force and provide the molecular mechanism of the initial complex formation.

Binding studies have relied on α-catenin co-sedimentation assays with F-actin, which are difficult to deconvolute because α-catenin tends to precipitate and because α-catenin transitions between a monomer and a dimer. Recent F-actin pull-down studies of monomeric α-catenin with amino-terminal deletions (Δ1–665, Δ1–670, Δ1–691, Δ1–695, and Δ1–698) and additional to Δ1–670, carboxy-terminal deletions (Δ873–906, Δ687–906, Δ865–906, Δ869–906) or a point mutation (W859A), showed affinities ranging from 0.4 µM (for Δ1–695) to 8.5 µM (for Δ1–664) with no binding detected for constructs that also had the carboxy terminus deleted (to just have residues 671–868 and 671–864) and over 35 µM affinity for the (residues 671–906) W859A mutant^35^. Since none of these truncated constructs dimerize, the results are difficult to put in the context of dimeric α-catenin. Also, the difficulty in reconciling the lack of binding of the residues 671–868 α-catenin construct compared to the four residues longer α-catenin construct (residues 671–872) that binds F-actin with 5 µM, with the cryoEM structures, was already noted becasue the four additional residues (869–872) are not observed in the cryoEM structures^35^. Indeed, the cryoEM maps for the truncated FABDs and our 2.8 Å map for our  residues 22-906 α-catenin sample allowed unambiguous tracing through residue 871. The carboxy terminus is also unstructured in the AlphaFold^37^ predicted structure and beyond residue 878 in our dimeric α-catenin crystal structure (PDB entry 4igg)^32^.

The amino-terminal flexibility of the α-catenin FABD upon binding was documented by limited proteolysis that was consistent with the noted weaker density for residues 699-702 in the earlier murine cryoEM structure of the isolated α-catenin FABD bound to F-actin^35^. Notably, for our Δ1–21 α-catenin sample, no density is visible before residue 710. While the isolated α-catenin FABD structures bound to F-actin suggested a weakly bound amino-terminal FABD α-catenin α-helix, this α-helix is severed from the FABD helical bundle in our 2.7 Å cryoEM structure of Δ1–21 α-catenin bound to F-actin. Given that (i) our Δ1–21 α-catenin bound to F-actin has residues amino-terminal of 710 disordered, (ii) in our monomeric α-catenin structure residues 699-710 engage in extensive contacts by being sandwiched between residues 631-635 that connect the middle domain to the linker α-helix 636–650, and (iii) in the truncated F-actin bound FABD structures, the cryoEM maps for residues amino-terminal of 710 are weak and challenging to assign, it seems that the first FABD α-helix plays a role far beyond regulating the affinity for F-actin. Additionally, the α-helix (residues 636–650) in our monomeric α-catenin structure is a random coiled region in the AlphaFold predicted structure. This suggests that this α-helix might act as one of the tension sensors.

Thus far, structural studies were limited to the α-catenin FABD or dimeric α-catenin, which leaves a void in our understanding of α-catenin monomeric specific functions. Our SEC-MALS data show, that deletion of the 81 amino-terminal α-catenin residues results in a purely dimeric protein that does not convert back into a monomer as was also shown for α-catenin Δ1–56^43^. Our cryoEM structure of the strictly dimeric α-catenin bound to the filamentous actin and our cryoEM structure of full-length monomeric α-catenin on its own provide important molecular clues into the functions of α-catenin in regulating cell adhesion. The first finding with important insights into how α-catenin regulates the cytoskeleton is the observation that the α-catenin monomer has its amino-terminal domain isolated from its middle domain. This flexibility is consistent with size exclusion chromatography small-angle X-ray scattering data that showed that the flexible linker from the middle domain leaves the amino-terminal domain flexible^38,39^.

Secondly, such lack of interdomain contacts is not the case for the carboxy-terminal domain, which is the FABD, of monomeric α-catenin, that engages in interactions with its middle domain that are not seen in our α-catenin dimer crystal structure^32^. Indeed, our monomeric α-catenin structure revealed the density corresponding to the linker that forms an α-helix that keeps the FABD close to the middle domain. Notably, the proximity of this α-helix to the first and to the last 4-helix bundle of the three middle α-catenin subdomains might restrict the FABD in the α-catenin monomer. We identified the first structural evidence of the linker region in our monomeric α-catenin structure. The central location of the linker α-helix and its position within the polypeptide chain, connecting the FABD to the middle domain, might be important in regulating the release of the FABD to bind to the actin cytoskeleton.

Third, in the absence of tension, the FABDs in our dimeric α-catenin bind F-actin without any interdomain α-catenin interactions. Collectively, our cryoEM structures and previously published data suggested that two α-helices keep monomeric α-catenin from binding F-actin efficiently while these two α-helices seem to serve as a spring to sever the interdomain interactions of the FABD in dimeric α-catenin. Thus, our F-actin-bound dimeric and unbound monomeric α-catenin structures seem to explain the constitutively active state of dimeric and the inefficient binding of monomeric α-catenin.

Vinculin is structurally and functionally closely related to α-catenin. However, vinculin is monomeric and does not dimerize. In contrast to α-catenin, vinculin has its FABD buried and autoinhibited^46–49^. The nanomolar affinity of the vinculin FABD with the vinculin amino-terminal domain correlates with the large buried surface area of the vinculin FABD of about 1800 Å^2^. In comparison, the FABD in our monomeric α-catenin cryoEM structure buries only about one-third (631 Å^2^) of the vinculin solvent accessible surface area. Our monomeric vinculin and α-catenin structures thus explain why only vinculin is autoinhibited.

**Fig. 5:**
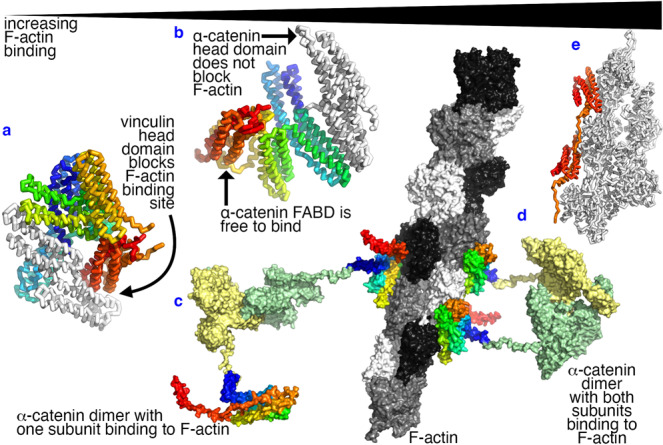
Bivalency and interdomain interactions as potential regulators of the binding of α-catenin to filamentous actin. **a** In vinculin, the vinculin head domain (white) binds to the F-actin binding site on the vinculin tail domain (colored from red to orange). Therefore, vinculin is autoinhibited with weak binding to F-actin. **b** In monomeric α-catenin, the F-actin binding domain (colored from red to orange) is accessible to bind to F-actin. Monomeric α-catenin might bind F-actin weaker as a monovalent protein. **c** The α-catenin dimer will likely have one of its FABDs binding to F-actin first. **d** Such initial interaction increases the avidity for the second FABD of the α-catenin dimer to bind to F-actin. **e** The minimal FABD (colored from red to orange) binds F-actin (white) with the highest affinity.

The fact that the FABDs reside on opposite sides in the α-catenin dimeric crystal suggests that this arrangement might facilitate dimeric α-catenin to crosslink two actin filaments^38^. However, the isolated FABD seems to bundle F-actin as shown by total internal reflection fluorescence microscopy^36^ suggesting that α-catenin dimerization is not necessary for actin bundling. Instead, dimerization might be an additional mechanism to explain that dimeric, but not monomeric, α-catenin binds F-actin readily in the absence of force given the mono- *versus* bivalent binding. Indeed, binding one α-catenin FABD to F-actin brings the second and unbound FABD in proximity to the unoccupied site on F-actin by tethering (Fig. 5). This proximity substantially increases the avidity, as for example, described for antibodies^50^, and would additionally explain why the binding of monomeric α-catenin without external load is weak^24,25,27^. While the cooperative binding of α-catenin to F-actin has been characterized previously^27,40^, avidity might be a new mechanism whereby dimeric α-catenin acts as a bivalent molecular switch. As discussed above, the newly discovered α-helical linker region might additionally contribute to the functional differences between the monomer and dimer. While less likely due to the proximity-increasing avidity reasons mentioned here, we do not rule out the possibility that only one FABD of the dimeric α-catenin binds F-actin.

Clearly, the binding of α-catenin to the actin cytoskeleton is key to cell adhesion. Our structures provide detailed knowledge and mechanistic insights on the interdomain flexibility of the multidomain α-catenin monomer and dimer. Such flexibility that we captured in the absence of force seems to correlate with α-catenin function as a mechanically sensitive adaptor. Thus, our data provide mechanistic insights into how the dimeric and monomeric α-catenin make initial contact with actin filaments. These events are crucial as they eventually lead to the remodeling of the actin cytoskeleton and the transition between cell migration and cell-cell adhesion. Two F-actin cryoEM studies^51,52^ corroborate our F-actin cryoEM structure and describe the solvent-mediated mechanism involving ATP in filament flexibility.

## Materials and methods

### Cloning and protein production

Human α-catenin (residues 22–906) was subcloned into a modified pET28 vector with an amino-terminal octa-histidine tag followed by a PreScission protease cleavage site. The set of primers used in the cloning of these constructs is as follows:

**Table:** 

Sample	Primer
pET28 vector	forward: TGAGAATTCGCGGCCGCACTCGAGCACCAC
	reverse: CATATGGGGCCCCTGGAACAGAACTTCCAGATG
human α-catenin (residues 22–906)	forward: GTTCTGTTCCAGGGGCCCCATATGACTCTGGCAGTTGAGAGACTGTTG
	reverse: CGGCCGCGAATTCTCAGATGCTGTCCATAGCTTTGAACTCGCTG

Full-length human α-catenin was cloned into pGEX-6P-1 with an amino-terminal glutathione S-transferase (GST)-tag and a PreScission protease cleavage site by DNA Custom Cloning, USA. All α-catenin constructs were transformed into BL21(DE3) (Novagen) and expressed in 2 liters of Luria-Bertani media at 37 °C until the optical density at 600 nm reached 0.6–0.8. Induction was performed with 1 mM isopropyl β-D-1-thiogalatopyranoside for 20 h at 25 °C. Cells were pelleted by centrifugation at 5000 × *g* for 15 min. Cell pellets were stored at −80 °C.

### Protein purification

To generate purified human α-catenin (residues 22–906), frozen *Escherichia coli* BL21(DE3) pellets containing amino-terminally His_8_-tagged α-catenin were thawed, resuspended in 500 mM NaCl, 20 mM Tris pH 8, and 1 mM β-mercaptoethanol. Cells were lysed by using a French Press. The lysate was clarified through centrifugation at 30,000 × *g* for 45 min and filtered. The supernatant was loaded onto two HisTrap HP 5 ml columns equilibrated in 500 mM NaCl, 20 mM Tris pH 8, and 1 mM β-mercaptoethanol, and connected in tandem to an AKTA FPLC at a flow rate of 1 ml/min. The column was then washed with a buffer containing 500 mM NaCl, 20 mM Tris pH 8, and 1 mM β-mercaptoethanol, followed by a second wash step with a buffer containing 500 mM NaCl, 20 mM Tris pH 8, 1 mM β-mercaptoethanol, and 100 mM imidazole. Elution was completed through a 200 ml gradient of buffer containing 500 mM NaCl, 20 mM Tris pH 8, 1 mM β-mercaptoethanol, and 500 mM imidazole. The elution peak was pulled and dialyzed overnight in 1 liter of a buffer containing 500 mM NaCl, 20 mM Tris pH 8, and 1 mM β-mercaptoethanol, after the addition of 200 μg of PreScission protease. The next day, the sample was loaded onto two HisTrap HP 5 ml columns equilibrated in 500 mM NaCl, 20 mM Tris pH 8, and 1 mM β-mercaptoethanol, which were connected in tandem at a 1 ml/min flow rate. The flowthrough containing cleaved α-catenin was collected, concentrated, and stored in 100 μl aliquots at −80 °C after adding 10% glycerol. For size exclusion chromatography, a 100 μl aliquot was thawed and loaded onto a Superdex S200 10/300 GL equilibrated in 150 mM NaCl, 20 mM HEPES pH 7.5, and 1 mM dithiothreitol. The protein eluted in two peaks, the first corresponding to dimeric α-catenin and the second to the monomeric α-catenin.

For the purification of full-length α-catenin, we thawed frozen *Escherichia coli* BL21(DE3) pellets containing GST-tagged α-catenin that we resuspended in lysis buffer (400 mM NaCl, 20 mM Tris pH 8, 0.5 mM ethylenediaminetetraacetic acid (EDTA), 2 mM β-mercaptoethanol and 1 mM phenylmethylsulfonyl fluoride). We lysed the cells by sonication, at 75% amplitude, for 3 min with 5 s and 10 s off cycles followed by centrifugation at 100,000 × *g* for 30 min at 4 °C. The clarified lysate was loaded onto a GST 16/10 Sepharose column that was equilibrated with 150 mM NaCl, 20 mM Tris pH 8, 0.5 mM EDTA, and 2 mM β-mercaptoethanol. We eluted the bound GST- α-catenin with 10 mM reduced glutathione. The peak fractions containing the GST-α-catenin were pooled, treated with 200 μg of PreScission protease, and dialyzed extensively against 2 liters of 150 mM NaCl, 20 mM Tris pH 8, 0.5 mM EDTA, and 1 mM β-mercaptoethanol. The dialyzed sample was then passed through a GST 16/10 Sepharose column, and the flowthrough fractions containing the GST-tag cleaved α-catenin were collected, concentrated, supplemented with 10% glycerol and flash frozen in liquid nitrogen as 500 μl aliquots until further processing. As a final step, the thawed aliquots were passed through a Superdex S200 10/300 GL column pre-equilibrated with 20 mM Tris pH 8, 150 mM NaCl, and 0.2 mM tris(2-carboxyethyl)phosphine (TCEP). The monomer and dimer peaks were pooled separately and loaded for a second time on Superdex S200 10/300 GL to get enriched monomer or dimer samples.

### Size exclusion chromatography coupled with multi-angle light scattering (SEC-MALS)

SEC-MALS was performed as described^53^. Briefly, to determine the absolute molar mass, SEC-MALS analyses were carried out using an Agilent 1260 Infinity HPLC system consisting of a variable wavelength detector for ultraviolet absorption monitoring coupled in line with Dawn-Heleos II multi-angle light-scattering detector (Wyatt Technology) and OptiLab T-rex differential refractive index detector (Wyatt Technology). Samples were passed through an analytical Superdex S200 10/300 GL column pre-equilibrated with 20 mM Tris-HCl pH 8, 150 mM NaCl, and 0.2 mM TCEP at a flow rate of 0.5 ml/min. Bovine serum albumin (BSA; from Pierce) was used as a standard, and all the data acquisition and subsequent analyses were carried out using ASTRA software version 6.1.

### Actin polymerization

Polymerization of F-actin was performed as described^53^. Briefly, rabbit skeletal muscle G-actin (50 μl at 1 mg/ml) was dialyzed against 500 ml of G-actin buffer (2 mM Tris pH 8.0, 0.2 mM adenosine triphosphate, 0.2 mM CaCl_2_, and 0.5 mM β-mercaptoethanol) overnight at 4 °C to ensure complete depolymerization of actin. G-actin (at 0.6 mg/ml) was then polymerized with the addition of polymerization buffer (50 mM KCl, 2 mM MgCl_2_, 10 mM imidazole, 2 mM ATP, and 5 mM EGTA) and left overnight to polymerize fully. F-actin prepared this way was stored at 4 °C and used for up to two weeks.

### Cryogenic electron microscopy grid preparation

For dimeric human α-catenin (residues 22–906) bound to F-actin, grids were prepared by reconstituting the complex on the grid. C-flat 1.2/1.3 400 mesh grids were glow discharged for 60 s and placed into a Leica GP2 cryogenic plunger set to 65% humidity and 21 °C. First, 3.5 μl of 0.7 μM F-actin diluted in 150 NaCl, 20 mM Tris pH 7.5, and 1 mM dithiothreitol was placed on the carbon film side of the C-flat grid and left to incubate for 1 min before the successive addition and removal of 3.5 μl of 7.6 μM (0.74 mg/ml) F-actin. The addition and removal of α-catenin was repeated four times before blotting from the backside of the grid. Grids were immediately plunged frozen in liquid ethane and transferred into a JEOL cryoARM300 for screening and data collection.

Vitrification of human monomeric α-catenin was carried out by applying 3 μl of 0.25 mg/ml α-catenin on a glow discharged 300 mesh Au-Flat holey grids (ProtoChips) with 1.3 μm holes at 95% humidity and 4 °C using a Leica GP2 plunge freezer. The blotting was carried out for 6 seconds, and the grids were immediately plunged frozen into liquid ethane. Frozen grids were then transferred into a JEOL cryoARM300 for screening and data collection.

### Cryogenic electron microscopy data collection

All datasets were collected on a JEOL cryoARM300 operated at 300 kV as described^53^. Briefly, movies were collected at a nominal magnification of 60,000 with parallel illumination with an Omega in-column energy filter tuned to a slit width of 20 eV. SerialEM^54^ (reference number to be corrected) was used to record electron micrographs using a K3 direct electron detector (Gatan) operated in correlated double sampling mode and a calibrated object pixel size of 0.72 Å with a corresponding flux of 7 electrons/pixel/second on the detector. Automatic alignment of zero loss peak is automatically carried out every 6 hours using the serialEM script.

For full-length human monomeric α-catenin data collection, the exposure time and dose rate were adjusted to provide a total dose of 48 electrons/Å^2^ *per* micrograph, fractionated into 40 frames to achieve a corresponding electron dose of 1.2 electrons/Å^2^ *per* frame. For all other samples, the exposure time and dose rate were adjusted to provide a total dose of 60 electrons/Å^2^ *per* micrograph, fractionated into 50 frames. High flashing of the cold field emission gun was set up through serialEM every 4 hours during data collection to maintain emission at nearly 10 µA. Astigmatism and coma-free alignments were performed with serialEM immediately before each data collection and repeated once a day for datasets lasting longer than one day.

For full-length human monomeric α-catenin, since Au-flat grids were used, astigmatism and coma-free alignments were performed using the carbon film of a C-flat grid that was briefly loaded before each data collection. For all datasets, micrographs were acquired from a pattern of 3 × 3 holes recording each image at the center of the hole. One focus step *per* stage shift was performed on the film area next to the central hole. Micrographs were collected with a targeted defocus of −0.6 to −2.6 μm, and gain-corrected movies were saved and transferred to a computing cluster for processing.

### Cryogenic electron microscopy image processing

CryoSPARC^55^ was used for the entire processing workflow of all datasets. Raw movies were imported directly into cryoSPARC, and motion correction was performed with Patch motion correction^56^ and a contrast transfer function (CTF) estimation with a CTF patch estimation. Curation of micrographs was performed to eliminate (i) all micrographs that had a CTF fit worse than 3.5 Å for the F-actin-contained samples or 5 Å for full-length human α-catenin, and a total full-frame motion distance larger than 150 pixels, (ii) micrographs that were astigmatic, and (**iii**) micrographs where thick or crystalline ice was detected. For all F-actin-containing samples, the Filament Tracer function was used to pick particles with relaxed criteria to pick as many particles as possible and remove unsuitable particles during the early 2D classification steps.

For α-catenin (residues 22–906) bound to F-actin, 25,442 micrographs were collected from four different grids to compensate for the partial or lack of decoration observed on many filaments. After micrograph curation, 7427 images were selected and processed further. Particles were extracted with a large box size of 1620 pixels (603 Å) to facilitate the separation of highly decorated from undecorated filaments through visual inspection. After extensive 2D classification, particles clustering in 2D classes with visibly high degrees of decoration were re-extracted with a smaller box size of 1080 pixels (402 Å), and 3D reconstruction was performed by using the helical refinement function of cryoSPARC^55^. The particles were then CTF refined twice with local CTF refinement^57^. A final round of helical non-uniform refinement^58^ was applied to lead to a 3D reconstruction of a map at 2.8 Å from 403,663 particles, with a helical twist of −166.7° and a helical rise of 27.5 Å (EMDB entry EMD-26772). From the same dataset, 2D classes with no evidence of decoration were separated after extensive 2D classification and used to reconstruct the unbound F-actin 3D map. Using the same processing steps as with the decorated particles, a map for unbound F-actin at 2.7 Å was achieved from 963,102 particles (EMDB entry EMD-26860), with a helical twist of −166.7°, and a helical rise of 27.4 Å.

For full-length human monomeric α-catenin, 14,333 micrographs obtained after curation were used for processing. A blob picker with a particle diameter of 80 to 120 Å was used for initial particle picking, and after curation, about 6 million particles were extracted with a box size of 360 pixels (266 Å) and further binned to 180 pixels (corresponding to a pixel size of 1.44 Å/pixel). After several iterative rounds of 2D classification, the resultant particle stacks were used to reconstruct multiple ab initio classes to remove non-ideal particles. The most promising ab initio class was subjected to further curation through additional rounds of 2D classification. The resulting particle stack was subjected to homogeneous and local CTF refinement to improve the overall features of the map. Non-uniform refinement was subsequently carried out to improve the resolution (EMDB entry EMD-27717). A final round of masked non-uniform refinement with a mask prepared from a flexibly fitted α-catenin middle domain and FABD of an AlphaFold model^37^ was carried out.

### Cryogenic electron microscopy structure determination

For determining the cryoEM structure of human α-catenin (residues 22-906) bound to F-actin, the 3.2 Å structure of the FABD of α-catenin bound to F-actin (PDB entry 6upv)^36^ was fitted into our 2.8 Å map using Chimera^59^ and initially refined with PHENIX^60,61^ using real-space refinement with simulated annealing^62,63^ to better accommodate our higher resolution map. The structure was then revised with COOT^64^. Despite using a low threshold and low-pass filter, α-catenin residues amino-terminal of amino acid 710 were not resolvable and not included. The density ends abruptly. There was also poor density for the short loop region connecting residues 804–811. Finally, there was no density for α-catenin residues carboxy-terminal of amino acid 871. For F-actin, residues amino-terminal of amino acid 5 and carboxy-terminal of amino acid 374 were not resolvable and not included. The remaining coordinates for both α-catenin and F-actin were visually inspected multiple times to improve complementarity through COOT^64^, and further refined through PHENIX^60,61^ by using rigid body real-space refinement, after which outliers were inspected and corrected. A total of six copies of F-actin and six copies of α-catenin were fitted into the map and the final structure was deposited with the Protein Data Bank (PDB entry 7utj).

For determining the cryoEM structure of unbound F-actin, the F-actin coordinates of our human α-catenin bound to F-actin (PDB entry 7utj) were fitted into the map by using Chimera^59^, after removal of the α-catenin coordinates. The structure was then initially refined with PHENIX^60,61^ by using real-space refinement with simulated annealing and then visually inspected multiple times to improve complementarity through COOT^64^, and further refined through PHENIX^60,61^ by using rigid body real-space refinement, after which outliers were inspected and corrected. The resulting structure was nearly identical to that of F-actin in our human α-catenin bound to the F-actin structure, except for a difference loop conformation (residues 45–50). The final F-actin structure at 2.7 Å was deposited with the PDB (entry 7uxf).

For determining the cryoEM structure of the full-length human monomeric α-catenin (residues 1–906), the human α-catenin AlphaFold model^37^ was used to fit into our final cryoEM map using Chimera^59^. The model was then further subjected to real-space refinement in COOT^64^.

### Statistics and reproducibility

Model analyses were carried out in Pymol (v. 2.5.2) and Chimera (1.14)^65^. Structural similarities were calculated, and variations reported as root mean square deviation were calculated using main-chain atoms with the “Super” command in Pymol.

### Reporting summary

Further information on research design is available in the Nature Portfolio Reporting Summary linked to this article.

## Supplementary information

Supplementary Information

Description of Additional Supplementary Files

Supplementary Data 1

Supplementary Data 2

Supplementary Data 3

Supplementary Data 4

Supplementary Data 5

Supplementary Data 6

Reporting Summary

## Data Availability

Final refined 3D reconstructed models were deposited in EMDB with accession codes EMD-26772 for human α-catenin bound to F-actin and EMD-26860 for unbound F-actin with corresponding PDB entries of 7utj and 7uxf, respectively. Monomeric α-catenin was deposited in EMDB with accession code EMD-27717. The plasmids for expression of full-length α-catenin and Δ1-21 a-catenin were deposited in Addgene with accession codes 186458 and 186460, respectively. Source data for Fig. 2 can be found in Supplementary Data 1-6.
