# Supplementary material for: Distinct inter-domain interactions of dimeric versus monomeric α-catenin link cell junctions to filaments
Source: Commun Biol. 2023 Mar 16;6:276. doi: 10.1038/s42003-023-04610-x (PMC10020564; doi:10.1038/s42003-023-04610-x)
Supplement: Supplementary file 1 — Supplementary Information [file 42003_2023_4610_MOESM1_ESM.pdf]

Supplementary Information for

Distinct inter-domain interactions of dimeric *versus* monomeric α-catenin link the membrane or junctions to filaments

Erumbi S. Rangarajan[1], Emmanuel W. Smith[1] & Tina Izard[1,2]*

[1] The Cell Adhesion Laboratory, UF Scripps, Jupiter, FL 33458 USA

[2] The Skaggs Graduate School, The Scripps Research Institute, Jupiter, FL 33458 USA

* candice.losey@ufl.edu

Figure S1. CryoEM data processing workflow for full-length human monomeric α-catenin

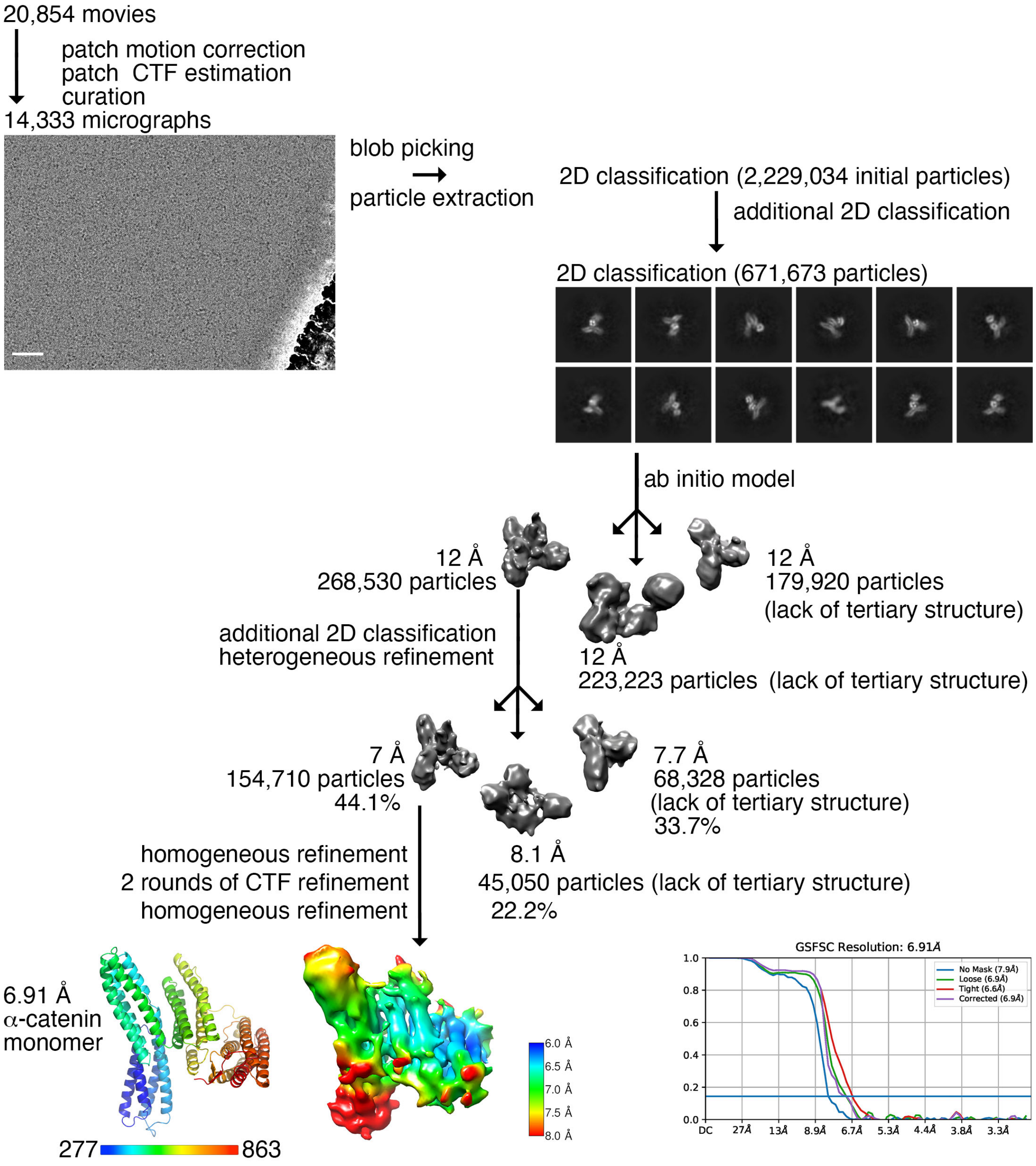

A representative image of the patch motion-corrected and patch CTF estimated micrograph is shown at the top. The scale bar on the micrograph corresponds to 50 nm. Representative 2D class averages of the particles used for structure determination are provided. Thumbnail images for various 3D volumes obtained at different stages are shown. The number of particles and resolution computed using two half-maps using a gold-standard Fourier shell correlation cut-off value of 0.143 are indicated. The 3D reconstruction of monomeric α-catenin resulted in a 6.9 Å resolution map.

Figure S2. The FABD of monomeric α-catenin engages in new interdomain interactions

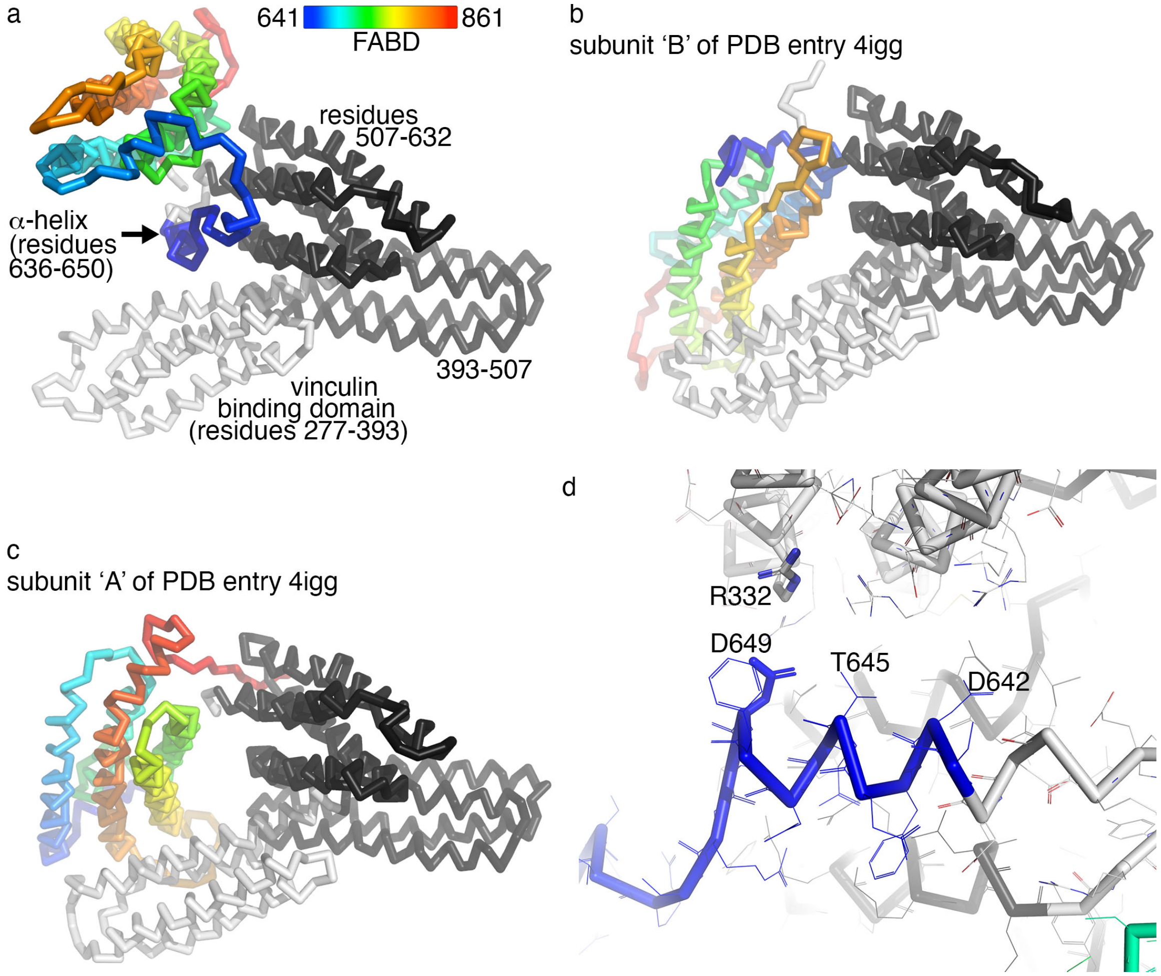

a Cα trace of our cryoEM structure of monomeric α-catenin. The middle domain is colored from black to white for residues 277 to 668. The carboxy-terminal F-actin binding domain (FABD) is colored spectrally from shorter to longer wavelengths as indicated for residue 641 to 861.

b Cα trace of subunit 'A' of the crystal structure of dimeric α-catenin (PDB entry 4igg)[1], color-coded and oriented as in panel (A). The amino-terminal domain is not shown.

c Cα trace of subunit 'B' of the crystal structure of dimeric α-catenin (PDB entry 4igg)[1], color-coded and oriented as in panel (A). The amino-terminal domain is not shown.

d New monomeric α-catenin interdomain interactions. The side chain representation is merely to show the characteristics of regions and their overall domain interaction and requires further confirmation through alternate approaches.

Figure S3. CryoEM data processing workflow for unbound F-actin and F-actin bound by dimeric α-catenin

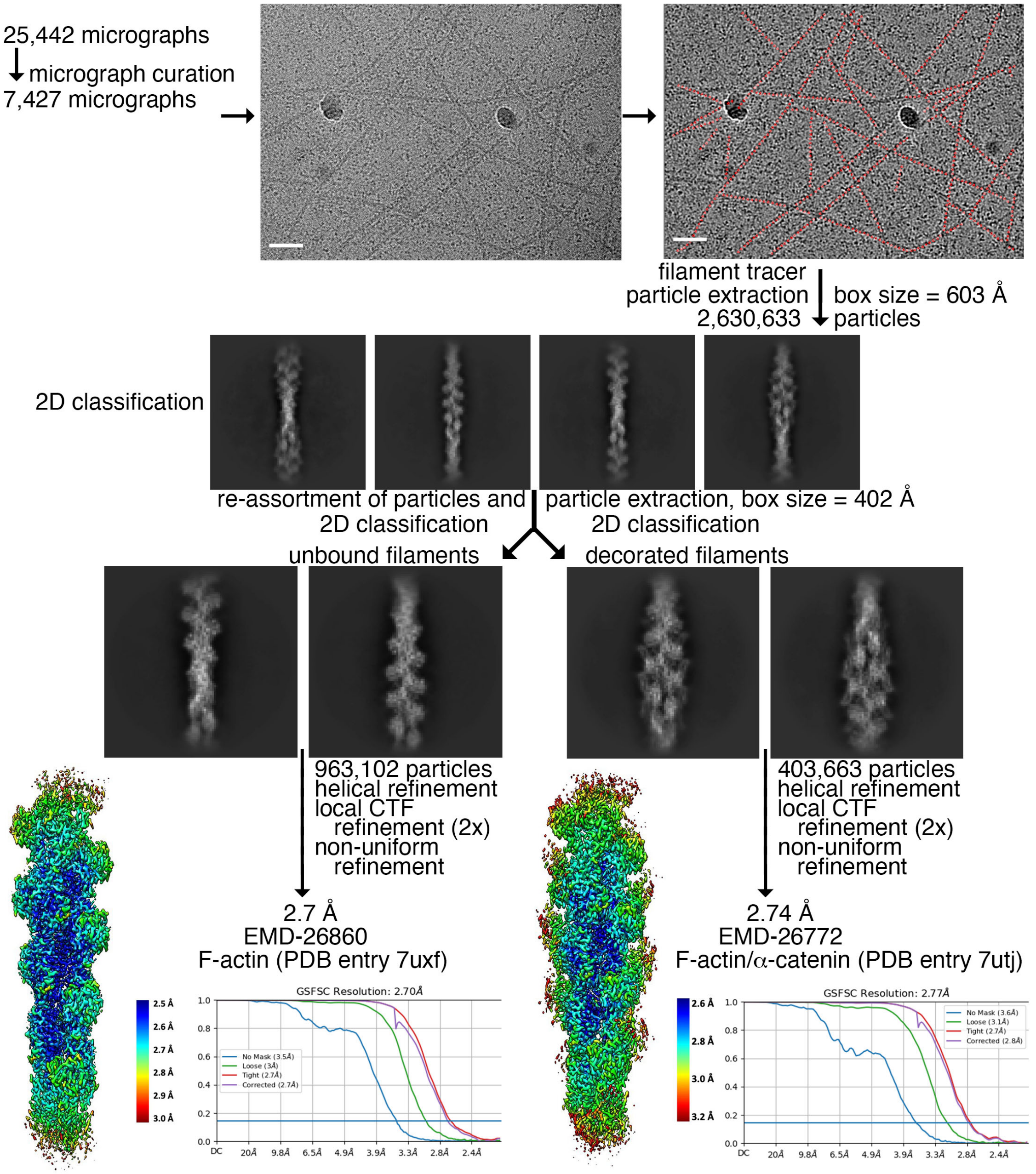

Representative micrographs are shown before (**top, left**) and after particle picking (**top, right**; red traces). Representative 2D classes of filaments (unbound and decorated, respectively) for F-actin bound by dimeric α-catenin are shown with a box size of 603 Å. The final 3D reconstruction workflow was the same for both maps resulting in a 2.7 Å resolution map for both unbound F-actin and F-actin bound by dimeric α-catenin, as estimated in cryoSPARC by Fourier shell correlation (FSC) with a cutoff at 0.143. The EMDB deposition identifiers, the fitted structures, and the corresponding PDB deposition identifiers are provided.

Figure S4. The 2.8 Å cryoEM structure of dimeric α-catenin bound to F-actin

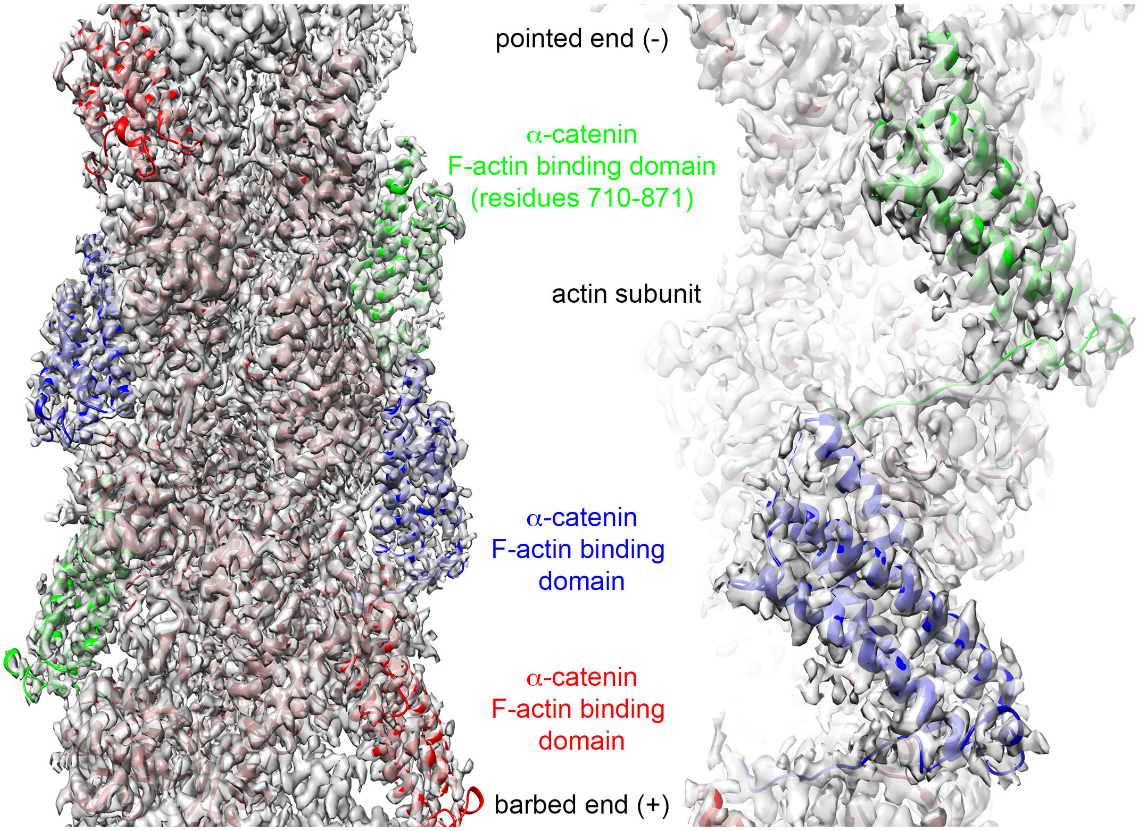

(**Left**) The refined coordinates of dimeric α-catenin bound to F-actin are overlayed onto the final 3D reconstruction (transparent grey surface). The actin subunits are colored brown while the F-actin binding domain of the dimeric α-catenin is colored red, green, or blue.

(**Right**) The structure of α-catenin bound to F-actin looking onto the carboxy-terminal interaction of the α-catenin FABD (in green) with an adjacent α-catenin FABD (in blue) overlaid onto the cryoEM map (transparent grey surface). The actin subunits are shown semi-transparent for clarity.

Figure S5. The carboxy-terminus of the FABD of dimeric α-catenin engages in new interdomain interactions

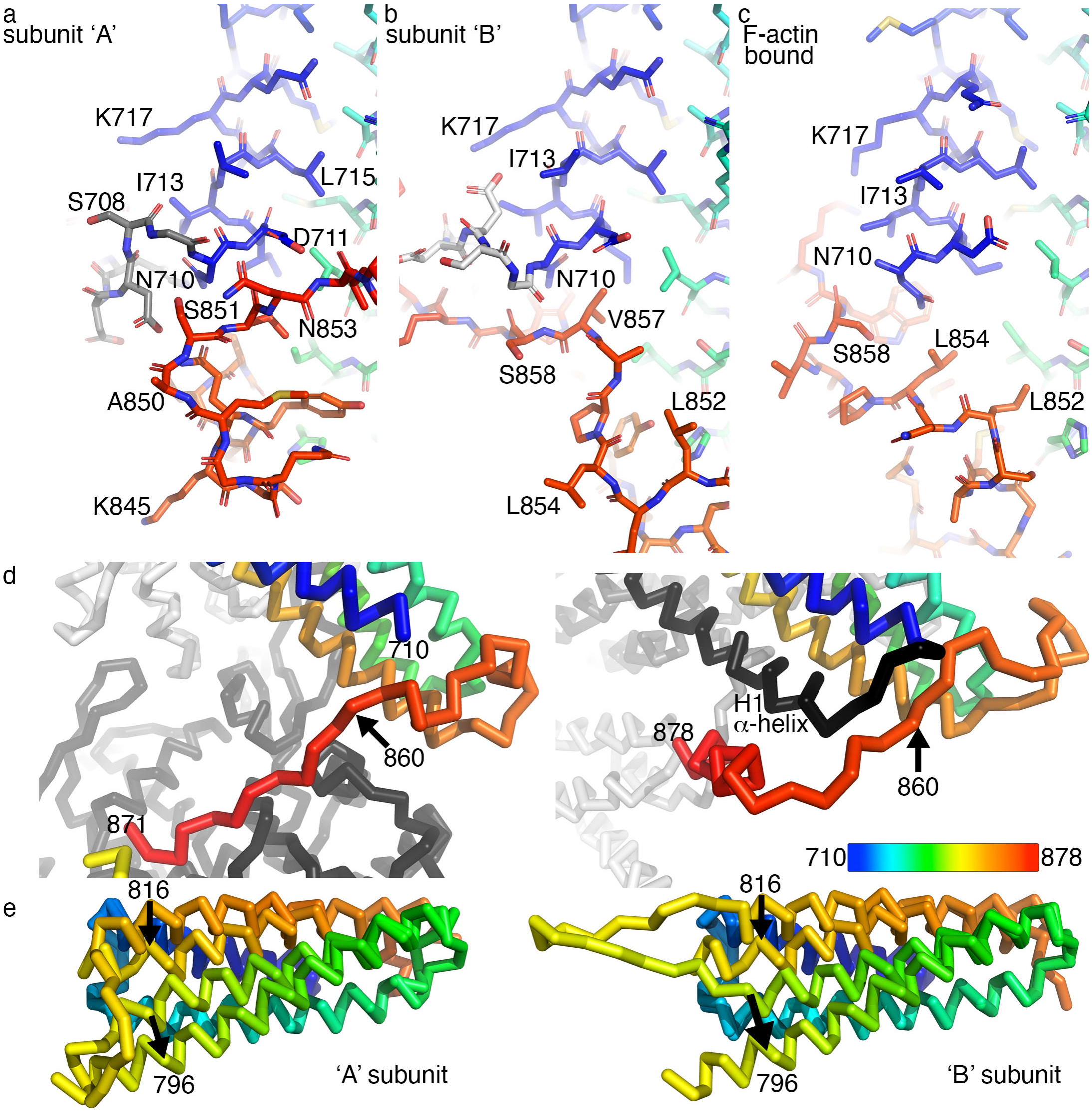

The carboxy-terminus (red) of the FABD interacting with the second α-helix (blue) of the 5-helix FABD bundle as seen in (**a**) subunit 'A' and (**b**) subunit 'B' of our dimeric α-catenin crystal structure (PDB entry 4igg)[1] or (**c**) in our F-actin bound dimeric α-catenin cryoEM structure. The orientation is the same in panels (a) through (c). Note the unique interaction of N710 residing at the amino terminus of the second α-helix (blue).

d Cα trace of (**left**) subunit 'B' of our crystal structure of dimeric α-catenin (PDB entry 4igg)[1] or of (**right**) our F-actin bound dimeric cryoEM α-catenin structure. The α-catenin FABD is colored spectrally as indicated. The first α-helix (**right**; 'H1') of the FABD in the unbound α-catenin structure is a disordered amino-terminal of residue 710 in the actin-bound structure (**left**) whereby the 860 region (arrow) fills the space of the first α-helix.

e Superposition Cα trace of (**left**) subunit 'A' or of (**right**) subunit 'B' of our crystal structure of dimeric α-catenin (PDB entry 4igg)[1] onto our F-actin bound dimeric cryoEM α-catenin structure. Note the movement (arrows) induced by the binding of F-actin for residues 796 (5.8 Å for 'A' and 8.6 Å for 'B') and 816 (4.7 Å for 'A' and 5 Å for 'B').

Table S1. Absolute molar mass determination by size exclusion chromatography coupled with multi-angle light scattering (SEC-MALS)

Individual peak fractions corresponding to human α-catenin monomer or dimer were pooled from previous size exclusion chromatography (SEC) runs and used for SEC-MALS analyses. The absolute molar mass was analyzed using Astra 6.0.

a The dimeric human α-catenin SEC pool has 13.8% of monomeric α-catenin reappearing on the SEC-MALS run.

b The monomeric human α-catenin SEC pool has 7.9% of dimeric α-catenin reappearing on the SEC-MALS run.

c The dimeric human α-catenin Δ1-21 SEC pool remains a dimer on the SEC-MALS run.

d The monomeric human α-catenin Δ1-21 SEC pool has 63.3% of dimeric α-catenin reappearing on the SEC-MALS run.

e The dimeric human α-catenin Δ1-81 SEC pool remains a dimer on the SEC-MALS run.

f The monomeric human α-catenin Δ1-81 SEC pool has 49.7% of dimeric α-catenin reappearing on the SEC-MALS run.

	**molar mass [kDa]**	**calculated molecular weight [kDa]**	**mass fraction**	**oligomer**
**a** dimeric α-catenin				
	122.2 ± 0.6%	100.30	0.138	monomer
	188 ± 0.3%	200.6	0.862	dimer
**b** monomeric α-catenin				
	93.3 ± 0.3%	100.30	0.921	monomer
	183.8 ± 0.3%	200.6	0.079	dimer
**c** monomeric α-catenin (Δ1-21)				
	111.7 ± 0.2%	100.65	0.367	monomer
	197.9 ± 0.3%	201.28	0.633	dimer
**d** dimeric α-catenin (Δ1-21)				
	191.4 ± 0.3%	201.28	1.00	dimer
**e** monomeric α-catenin (Δ1-81)				
	98.9 ± 0.4%	92.08	0.503	monomer
	175.3 ± 0.3%	184.16	0.497	dimer
**f** dimeric α-catenin (Δ1-81)				
	178.3 ± 0.3%	184.16	1.00	dimer

Table S2. Possible interactions within 5 Å between the α-catenin middle domain and the α-catenin F-actin binding domain (FABD) as seen in monomeric α-catenin

Interactions as observed between the α-catenin middle domain (residues 275-634) and the α-catenin FABD (residues 660-906) were observed by interface analyses using PISA and PIC servers[2,3].

a hydrophobic interactions

**α-catenin FABD**		**α-catenin middle domain**	
position	residue	position	residue
714	Val	568	Try
714	Val	631	Met
714	Val	632	Ile
759	Leu	567	Val
857	Val	567	Val

b polar interactions

**α-catenin FABD**		**α-catenin middle domain**	
position	residue	position	residue
711	Asp	567	Val
718	Gln	571	Lys
858	Ser	564	Glu

c ionic interactions

**α-catenin FABD**		**α-catenin middle domain**	
position	residue	position	residue
860	Lys	564	Glu

Table S3. Intermolecular interactions for the α-catenin/F-actin complex as calculated with PISA[2]

a hydrogen bonding interactions

**α-catenin**	**[Å]**	**actin**
ARG 782 [NH2]	3.57	MET 47 [O]
ARG 782 [NH2]	3.25	TYR 53 [OH]
TYR 837 [OH]	3.52	ARG 95 [O]
LYS 866 [NZ]	3.22	ASP 25 [OD2]
LYS 867 [N]	3.10	PRO 27 [O]
CYS 772 [SG]	3.62	HIS 87 [NE2]
ASP 775 [OD1]	2.92	LYS 50 [NZ]
TYR 779 [OH]	3.03	LYS 50 [NZ]
MET 861 [SD]	3.27	ARG 95 [NH1]
LYS 862 [O]	2.58	ARG 95 [NH2]
PRO 864 [O]	3.19	ARG 28 [NH2]

**α-catenin**	**[Å]**	**neighboring actin**
LYS 795 [NZ]	3.13	GLY 23 [O]
CYS 793 [SG]	3.19	ALA 144 [O]
ASN 824 [ND2]	3.46	THR 148 [OG1]
LYS 797 [NZ]	2.93	GLU 334 [OE2]
HIS 788 [NE2]	2.43	SER 348 [OG]
ASP 813 [OD2]	3.84	ALA 331 [N]

b ionic interactions

**α-catenin**	**[Å]**	**actin**
LYS 797 [NZ]	3.90	GLU 334 [OE1]
LYS 797 [NZ]	2.93	GLU 334 [OE2]

**α-catenin**	**[Å]**	**neighboring actin**
LYS 866 [NZ]	3.72	ASP 25 [OD1]
LYS 866 [NZ]	3.22	ASP 25 [OD2]
ASP 775 [OD1]	2.92	LYS 50 [NZ]

SI References

1 Rangarajan, E. S. & Izard, T. Dimer asymmetry defines α-catenin interactions. *Nature Structural & Molecular Biology* **20**, 188-193, doi:10.1038/nsmb.2479 (2013).

2 Krissinel, E. & Henrick, K. Inference of macromolecular assemblies from crystalline state. *J Mol Biol* **372**, 774-797, doi:10.1016/j.jmb.2007.05.022 (2007).

3 Tina, K. G., Bhadra, R. & Srinivasan, N. PIC: Protein Interactions Calculator. *Nucleic Acids Res* **35**, W473-476, doi:10.1093/nar/gkm423 (2007).
