# Supplementary material for: Distinct inter-domain interactions of dimeric versus monomeric α-catenin link cell junctions to filaments
Source: Commun Biol. 2023 Mar 16;6:276. doi: 10.1038/s42003-023-04610-x (PMC10020564; doi:10.1038/s42003-023-04610-x)
Supplement: Supplementary file 2 — Description of Additional Supplementary Files [file 42003_2023_4610_MOESM2_ESM.docx]

**Description of Additional Supplementary Files**

File name: Supplementary Data 1

Description: The source data for graph in

Figure 2A.

File name: Supplementary Data 2

Description: The source data for graph in

Figure 2B.

File name: Supplementary Data 3

Description: The source data for graph in

Figure 2C.

File name: Supplementary Data 4

Description: The source data for graph in

Figure 2D.

File name: Supplementary Data 5

Description: The source data for graph in

Figure 2E.

File name: Supplementary Data 6

Description: The source data for graph in

Figure 2F.
