# Supplementary material for: Distinct inter-domain interactions of dimeric versus monomeric α-catenin link cell junctions to filaments
Source: Commun Biol. 2023 Mar 16;6:276. doi: 10.1038/s42003-023-04610-x (PMC10020564; doi:10.1038/s42003-023-04610-x)
Supplement: Supplementary file 9 — Reporting Summary [file 42003_2023_4610_MOESM9_ESM.pdf]

nature portfolio

Corresponding author(s): Smith, Rangarajan, Izard

Last updated by author(s): Jan 24, 2023

Reporting Summary

Nature Portfolio wishes to improve the reproducibility of the work that we publish. This form provides structure for consistency and transparency in reporting. For further information on Nature Portfolio policies, see our Editorial Policies and the Editorial Policy Checklist.

Statistics

For all statistical analyses, confirm that the following items are present in the figure legend, table legend, main text, or Methods section.

n/a	Confirmed	
☒	☐	The exact sample size (*n*) for each experimental group/condition, given as a discrete number and unit of measurement
☒	☐	A statement on whether measurements were taken from distinct samples or whether the same sample was measured repeatedly
☒	☐	The statistical test(s) used AND whether they are one- or two-sided<br>*Only common tests should be described solely by name; describe more complex techniques in the Methods section.*
☒	☐	A description of all covariates tested
☒	☐	A description of any assumptions or corrections, such as tests of normality and adjustment for multiple comparisons
☒	☐	A full description of the statistical parameters including central tendency (e.g. means) or other basic estimates (e.g. regression coefficient) AND variation (e.g. standard deviation) or associated estimates of uncertainty (e.g. confidence intervals)
☒	☐	For null hypothesis testing, the test statistic (e.g. *F*, *t*, *r*) with confidence intervals, effect sizes, degrees of freedom and *P* value noted<br>*Give P values as exact values whenever suitable.*
☒	☐	For Bayesian analysis, information on the choice of priors and Markov chain Monte Carlo settings
☒	☐	For hierarchical and complex designs, identification of the appropriate level for tests and full reporting of outcomes
☒	☐	Estimates of effect sizes (e.g. Cohen's *d*, Pearson's *r*), indicating how they were calculated

Our web collection on statistics for biologists contains articles on many of the points above.

Software and code

Policy information about availability of computer code

Data collection	SerialEM was used for cryoEM data collection.<br>SEC-MALS data was collected using Agilent HPLC system connected with Wyatt's DAWN HELEOS-2 and Optilab T-rex modules.
Data analysis	The software used for structure determination and analysis includes CryoSPARC-v-3.3.1, UCSF Chimera 1.14, Phenix1.18, Coot-0.9 and Pymol 2.4.

For manuscripts utilizing custom algorithms or software that are central to the research but not yet described in published literature, software must be made available to editors and reviewers. We strongly encourage code deposition in a community repository (e.g. GitHub). See the Nature Portfolio guidelines for submitting code & software for further information.

Data

Policy information about availability of data

All manuscripts must include a data availability statement. This statement should provide the following information, where applicable:

- Accession codes, unique identifiers, or web links for publicly available datasets
- A description of any restrictions on data availability
- For clinical datasets or third party data, please ensure that the statement adheres to our policy

The final refined electron microscope density maps generated and analyzed during the current study have been deposited in EMDB and PDB with accession codes EMD-26860 and PDB ID 7uxf for Apo F-actin, EMD-26772 and PDB ID 7utj for alpha-catenin:F-actin complex, and EMD 27717 for alpha-catenin monomer.

Human research participants

Policy information about studies involving human research participants and Sex and Gender in Research.

Reporting on sex and gender	Current study does not involve human research participants.
Population characteristics	Current study does not involve human research participants.
Recruitment	Current study does not involve human research participants.
Ethics oversight	Current study does not involve human research participants.

Note that full information on the approval of the study protocol must also be provided in the manuscript.

Field-specific reporting

Please select the one below that is the best fit for your research. If you are not sure, read the appropriate sections before making your selection.

☒ Life sciences ☐ Behavioural & social sciences ☐ Ecological, evolutionary & environmental sciences

For a reference copy of the document with all sections, see nature.com/documents/nr-reporting-summary-flat.pdf

Life sciences study design

All studies must disclose on these points even when the disclosure is negative.

Sample size	SEC-MALS analysis were carried out in duplicates. CryoEM data involves collection of data over multiple sessions for proper processing and analyses.
Data exclusions	Curation of micrographs was performed to eliminate (i) all micrographs that had a CTF fit worse than 3.5 Å for the F-actin-contained samples or 5 Å for full-length human alpha-catenin, and a total full-frame motion distance larger than 150 pixels, (ii) micrographs that were astigmatic, and (iii) micrographs where thick or crystalline ice was detected.
Replication	SEC-MALS analysis were carried out in duplicates.
Randomization	Randomization is not relevant to this study as no separate statistical analysis were performed.
Blinding	Blinding is not applicable to this study as the source of protein was known already.

Reporting for specific materials, systems and methods

We require information from authors about some types of materials, experimental systems and methods used in many studies. Here, indicate whether each material, system or method listed is relevant to your study. If you are not sure if a list item applies to your research, read the appropriate section before selecting a response.

Materials & experimental systems

n/a	Involved in the study
☒	☐ Antibodies
☒	☐ Eukaryotic cell lines
☒	☐ Palaeontology and archaeology
☒	☐ Animals and other organisms
☒	☐ Clinical data
☒	☐ Dual use research of concern

Methods

n/a	Involved in the study
☒	☐ ChIP-seq
☒	☐ Flow cytometry
☒	☐ MRI-based neuroimaging
